# Evaluation of replicas manufactured in a 3D-printed nanoimprint unit

**DOI:** 10.3762/bjnano.9.149

**Published:** 2018-05-28

**Authors:** Manuel Caño-García, Morten A Geday, Manuel Gil-Valverde, Xabier Quintana, José Manuel Otón

**Affiliations:** 1 CEMDATIC, ETSI Telecomunicación, Universidad Politécnica de Madrid, Av. Complutense 30, 28040-Madrid, Spain

**Keywords:** nanoimprint, oriented gradient, photoresist, polymer, replica

## Abstract

Nanoimprint lithography has become a useful tool to prepare elements containing nanoscale features at quite reasonable cost, especially if the fabrication elements are created in the own laboratory. We have designed and fabricated a whole nanoimprint manufacturing system and analyzed the resulting surfaces using ad hoc packages developed on an open-software AFM image analysis suite. To complete the work, a number of polymers have been thoroughly studied in order to select the best material for this implementation. It turned out that the best alternative was not always the same, but depended on the application. A comparative study of the polymers, which takes into account the values and dispersion of numerous sample parameters, has been carried out. As a large number of samples was prepared, an automatized procedure for characterization of nanoimprint surfaces had to be set up. The procedure includes figures of merit for comparative purposes. Materials without the requirement of a solvent were found to be superior for most nanoimprint applications. A large dispersion of the samples was found.

## Introduction

Although the basic principles behind nanoimprint lithography (NIL) have been known for many years, NIL was specifically mentioned for the first time by Stephen Y. Chou in his classic work [1], in which he named the technique and demonstrated a lithographic resolution of under 100 nm, by physical deformation of a thermoplastic polymer. Since then, NIL has proved to be a simple, reliable, low-cost, high-resolution and high-throughput technique for manufacturing nanoscale patterns. At present, there are a number of NIL methods, including thermal printing, UV curing, and roll-to-roll transfer [2], and NIL has found applications in many dissimilar fields such as standard semiconductor microelectronics, optics, photonic integrated circuits, plasmonics or microfluidics [3].

NIL is customarily split into three categories, “hard”, “soft” and “hybrid” NIL [4], depending on the kind of mold employed in the transfer. Hard NIL uses molds made of quartz or silicon, and allows for the transfer of features below 10 nm, but increases significantly the defect rate from contaminating particles and air bubbles. Soft NIL employs molds of elastomeric materials such as polydimethylsiloxanes (PDMS) or polyimides at the expense of lower resolution [5]. Hybrid NIL adopts an intermediate position by utilizing UV-curable molds. In either case, the samples can be used as such, or undergo further processes such as chemical etching [6] or electric-field-assisted steps [7].

In recent months, we have developed a NIL system [8] using free software and public hardware designs created in a simple 3D printer. The purpose was to obtain a reliable instrument at minimum cost, with the lowest reduction in performance. To test reliability and performance of the unit, a large number of samples made of different polymer materials have been prepared.

The purpose of this work is twofold: On one hand, given a set of specifications, we are trying to find the best polymers for nanoimprint; on the other hand, we intend to establish a characterization protocol for materials evaluation capable of discriminate valid materials for those specifications, e.g., comparison with theoretical values and dispersion of measurements. Hence a comparative procedure has been included to allow for a formal ranking of tested materials.

## Qualifying Procedures

Qualifying the suitability of any given material depends on the issues that are considered relevant. In our case, the main aim was to qualify the manufacturing process and how it applies to different materials. Therefore parameters widely employed for surface-quality analysis of micro- and nano-surfaces, such as kurtosis or skewness [9], are of limited interest in this context. Within the characterization protocol we have proposed a number of parameters, and have implemented specific procedures for measuring these magnitudes. The underlying clue is to gather some simple numbers qualifying the samples. The original idea was to propose a single figure of merit, but this turned out unfeasible, since many parameters are derived from numerical data through image processing of an AFM software package. Moreover, the proposed parameters often rely on the specific master (saw-tooth grating) employed in these series. Comparison between these parameters and classical measurements is not trivial and may induce false evaluations of the materials performance. It was therefore decided to complement the single figure of merit with other figures referring to specific aspects of manufacturing.

The parameters obtained from direct measurements are physical dimensions of the grating: pitch, blazing angle and back angle, i.e., the angle opposite to the blazing angle. The remaining parameters proposed in this work, taken from AFM image processing, are a map of RMS differences from theoretical to actual heights, a histogram of oriented gradients (HOG), the average height, and the average height-to-pitch ratio. All these parameters are explained in the next sections.

## Numerical Parameters

Support software of AFM units has become a sophisticated tool from which a high volume of numerical and graphical information can be appraised. Many packages, moreover, are open software, thus processing a high number of raw data may ultimately result in a relatively quick and simple task.

**Figure 1:**
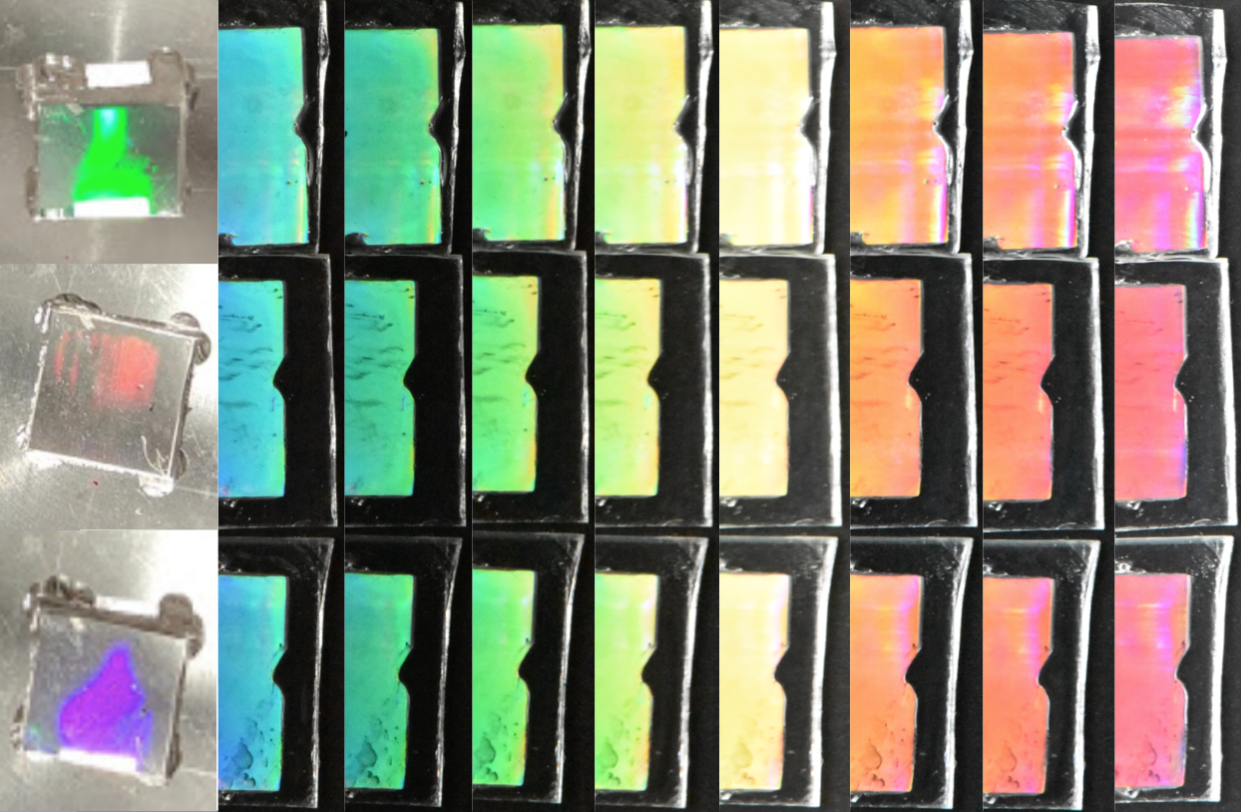
Two-step fabrication of samples. The left column shows three pictures taken at different angles of a negative PDMS mold created from the master. The remaining columns show three replicas (one per row) at different angles obtained by nanoimprint from the PDMS mold.

An example of macroscopic results is shown in Figure 1. Measurements were postprocessed using two scripts successively. The first script processes raw AFM data, obtaining the fundamental numerical parameters – pitch, blazing angle and back angle – mentioned below, as well as a BMP graphic file containing either the surface or a transformation of the same that will be eventually processed by the second script. The first script was written in Python language, through a virtual Pygwy console of a Gwyddion package [10]. Gwyddion is a software environment specifically designed for data analysis of scanning probe microscopy.

**Pitch:** A saw-tooth pattern shows a spatial frequency that can be checked after the imprinting, to ascertain whether the pitch is kept or some shrinking or lengthening has arisen during the curing process. The pitch variation and its dispersion for every tested material must be known. To fully take into account the 2D nature of the topography, 2D fast Fourier transform was applied on to obtain the spatial frequency. The pitch value is obtained from the inverse distance between the zeroth order and the first order of the FFT (actually the script takes the distance between the two first orders and divides by two). In practice this is equivalent to average pitch estimations taken from different 1D profiles along the sample width. A material is given the highest quality score in this parameter if it shows the same pitch as the master. The lowest score is given to samples having zero pitch or twice the master pitch. Intermediate values are linearly distributed.

**Scale maximum:** Scale maximum is not employed as a characterizing parameter because it is not actually the average of pattern maxima, but simply the highest value found in the pattern. The value is directly captured in the script just finding the maximum value. It gives an approximate idea of the image scale and is used for certain general calculations within the code.

**Figure 2:**
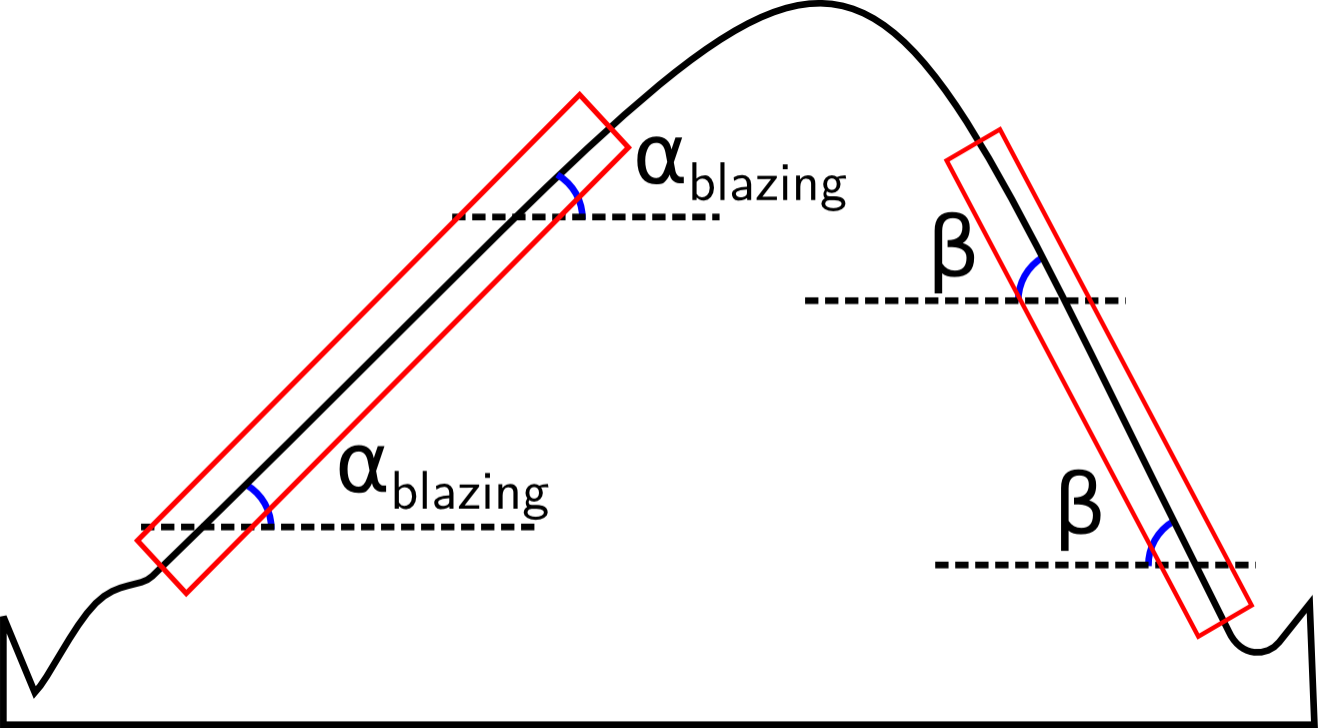
2D saw-tooth sketch showing the blazing angle α and the back angle β. Measurements are confined to the linear area of the slopes (red rectangles), where the angles are supposedly constant.

**Angles:** The saw-tooth pattern is characterized by its pitch and angles. The blazing angle, α_blazing_, determines the slope of the sawtooth and ultimately the sample topography. The blazing angle and the opposite back angle, β, are considered essential to estimate the quality of the copy. To evaluate the quality, the angles obtained from the copies are directly compared to the angles taken from the measurements of the master. Only the linear parts are considered in the calculation, as shown in Figure 2. Analogously to the pitch case, the mean master angle is considered the maximum achievable value. The lowest value is 0°.

## Image Processing Parameters

**Figure 3:**
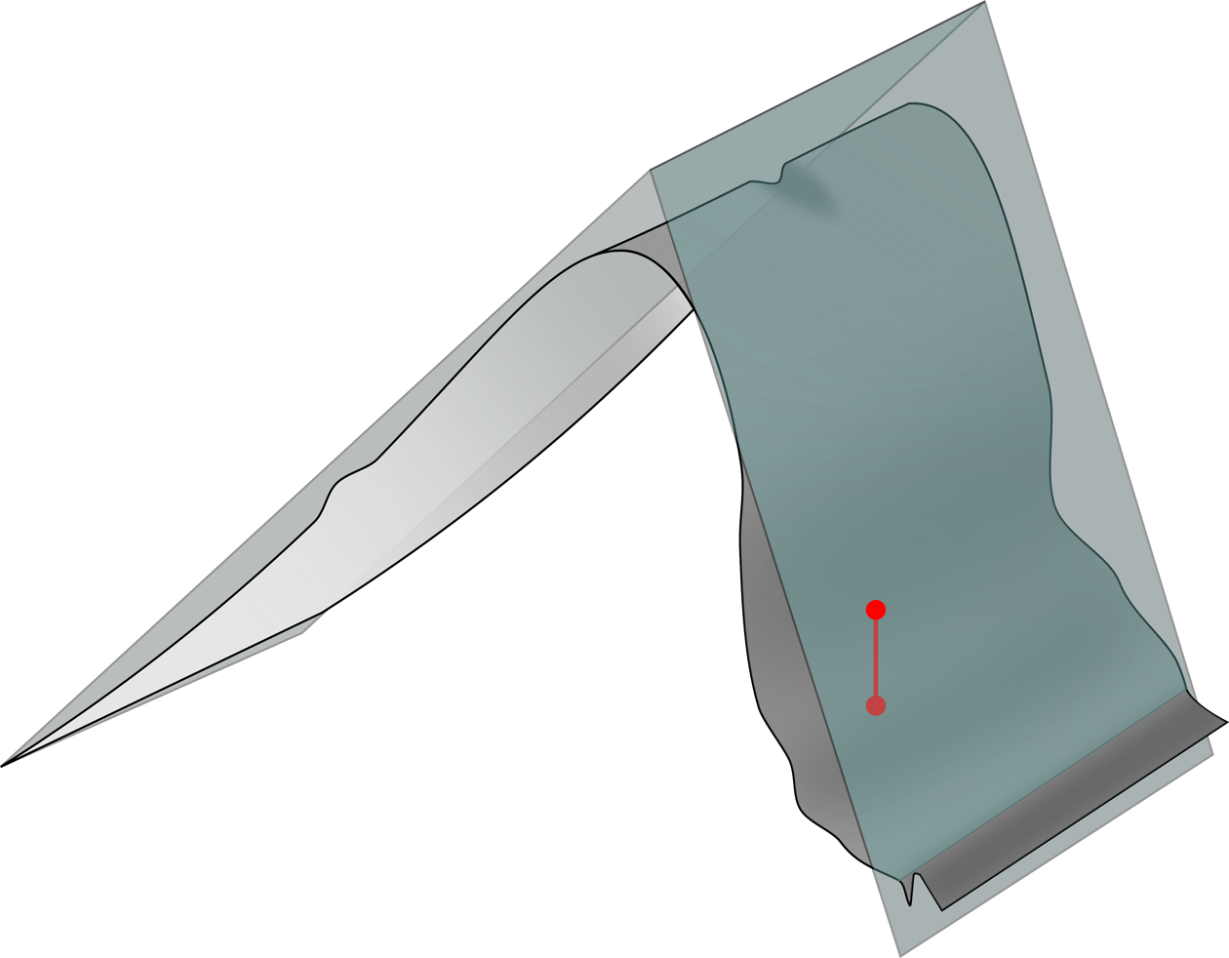
3D sketch of a real saw-tooth copy compared to its theoretical master. The vertical difference between the mesh points of these two curves (red segment) is used to evaluate RMS.

The second script is written and executed on a MatLab interface employing the MatLab Computer Vision Toolbox. It utilizes the output of the Python script to evaluate the second set of parameters, those derived from graphical inputs as described above:

**Vertical RMS deviations:** Every copy is compared to a perfect saw-tooth pattern having the same pitch and blazing/back angles. For every point of the 512 × 512 2D mesh, the height of the experimental points is compared to the theoretical saw-tooth pattern (Figure 3). The root mean square (RMS) deviation is calculated as

[1]
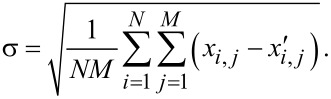


**Figure 4:**
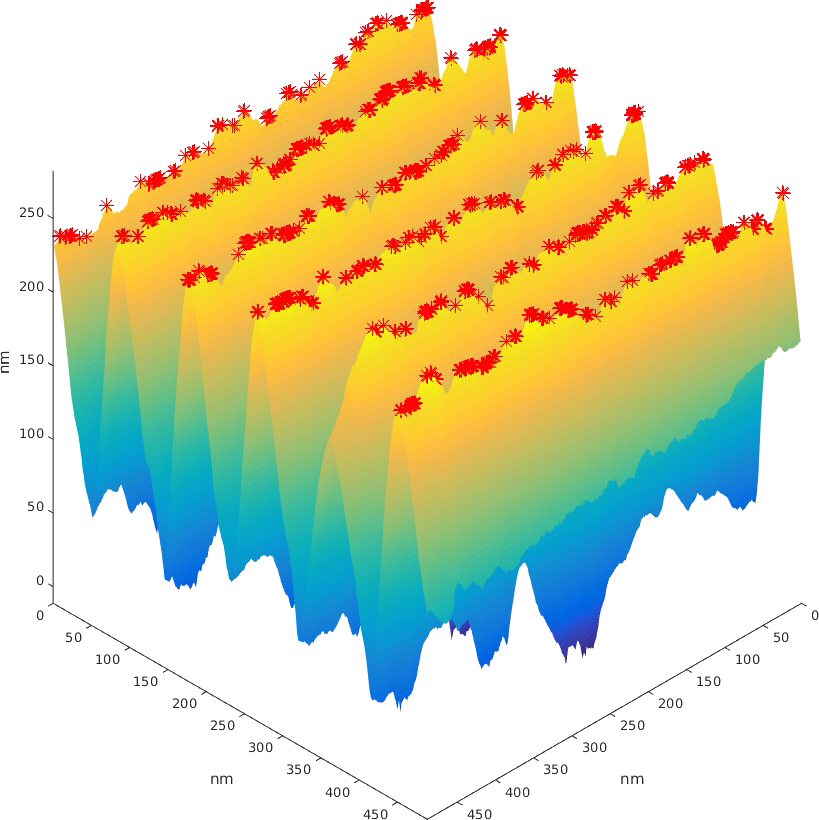
Plot of an actual saw-tooth copy showing the average height maxima (red points).

**Average height:** The MatLab script also includes a calculation of the average height. The aim is not to find maximum height values, but averaged local maxima over a certain area that can be adjusted through a parameter in the script (Figure 4). The purpose is to avoid glitches, ratches and other artifacts in the evaluation of height values.

**Height-to-pitch ratio:** This parameter is useful to analyze whether pitch variations in the samples is due to a volume shrinkage/expansion or just a surface stretching. In the first case, the features are retained but with a different size, while in the second case some relevant features may have been affected by deformations.

**Figure 5:**
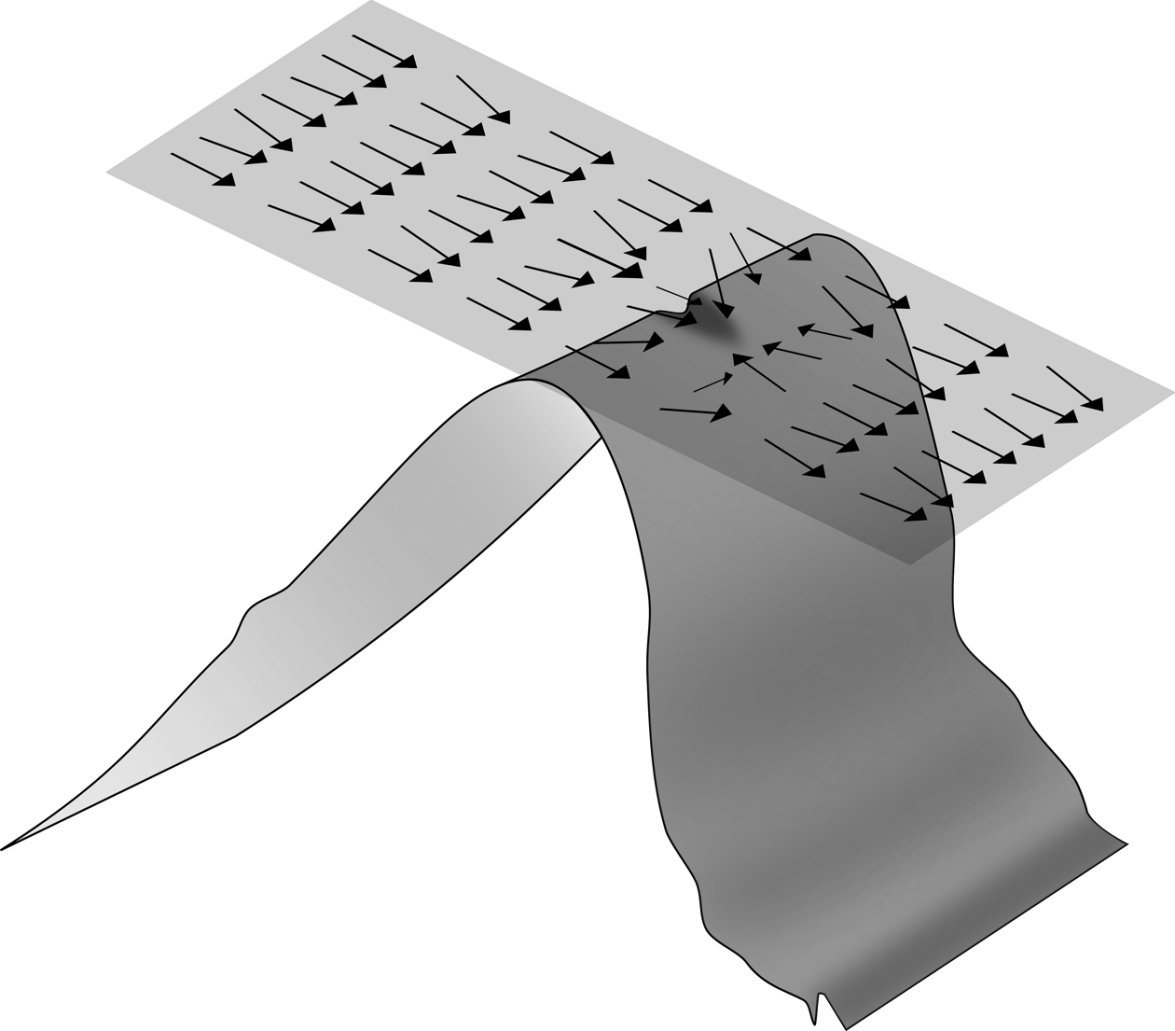
Calculation of HOG descriptor for a given surface. Vectors points to the direction of highest gradient; this should be a parallel set of vectors if the sample was perfect. Deviations from this direction show the presence of defects. See text for details.

**HOG parameter:** The histogram of oriented gradients (HOG) is a feature descriptor widely used in image processing. First described a decade ago [11], it is usually employed to detect objects in images, especially pedestrians. Here we propose to apply HOG to quantify the amount of superficial errors of a given topography. The underlying concept is relatively simple: In the 2D mesh of the sample under analysis, the maximum change rate (gradient) of different points (pixels) over a mesh is obtained and their directions are marked with vectors (Figure 5). These vectors include information about their direction (the sense is not relevant) and their moduli. In our case a saw-tooth is being reproduced; if it was perfect, the copy should show a set of vectors parallel to the grating pitch over the slope and back angle. However, the vectors show up divergences depending on the material and the manufacturing procedure. The number and magnitude of such imperfections can be related to the quality of the copy. The result is a single multicomponent vector (typically, several thousands of elements) called HOG descriptor, which is assigned to every image, i.e., the whole image information is condensed in one result. This is quite convenient for comparisons between samples, but data elaboration is involved. Fortunately, the MatLab Computer Vision Toolbox contains a set of functions to calculate all relevant parameters related to HOG, making it much simpler to prepare a script that gathers the required information.

**Figure 6:**
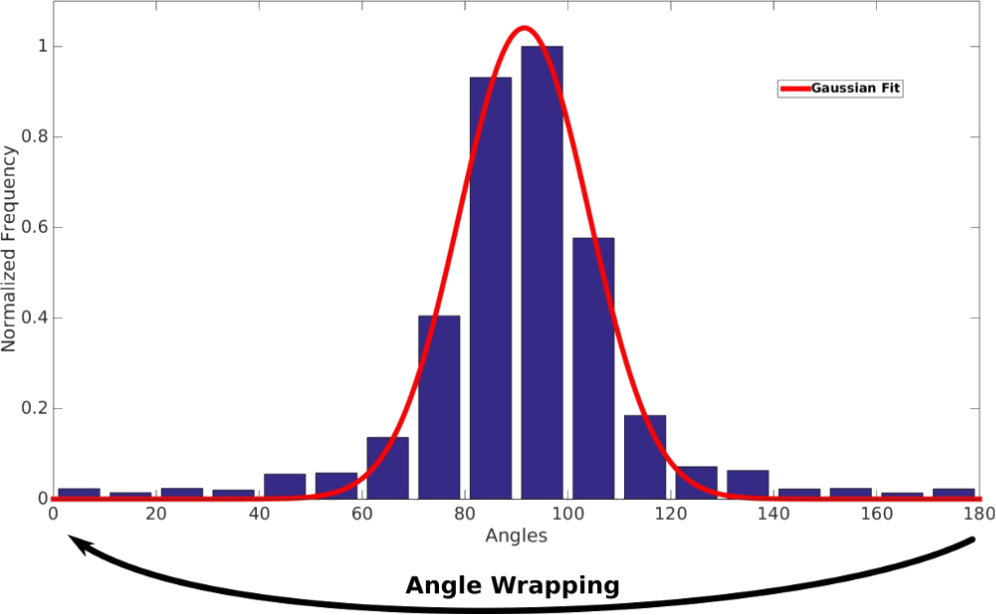
Calculation of HOG descriptor. The histogram corresponding to the pixels of one cell. A 17-bin histogram of 0–170° is used. Gradients of every pixel are accumulated in the columns; the angles wrap around, i.e., 180° is equivalent to 0°. The histogram is fitted using a normal Gaussian distribution, and the Gaussian FWHM is computed.

First, the gradients of a chosen point (actually, every pixel) of the surface with respect to its closest neighbors are calculated. In our case, the magnitude is taken from the grayscale (0–255) according to the gray level, and the direction depends on the position of the neighbor. Pixels are grouped into cells; each cell is associated to either a 9-bin histogram corresponding to angles of 0–160° or a 17-bin histogram of 0–170°. The histogram slabs accumulate the gradients included in each angle range (Figure 6). For any given gradient, its magnitude is proportionally distributed between the previous and the next bin. As mentioned above, the vector sense is not relevant, i.e., 180° is equavialent to 0°.

**Figure 7:**
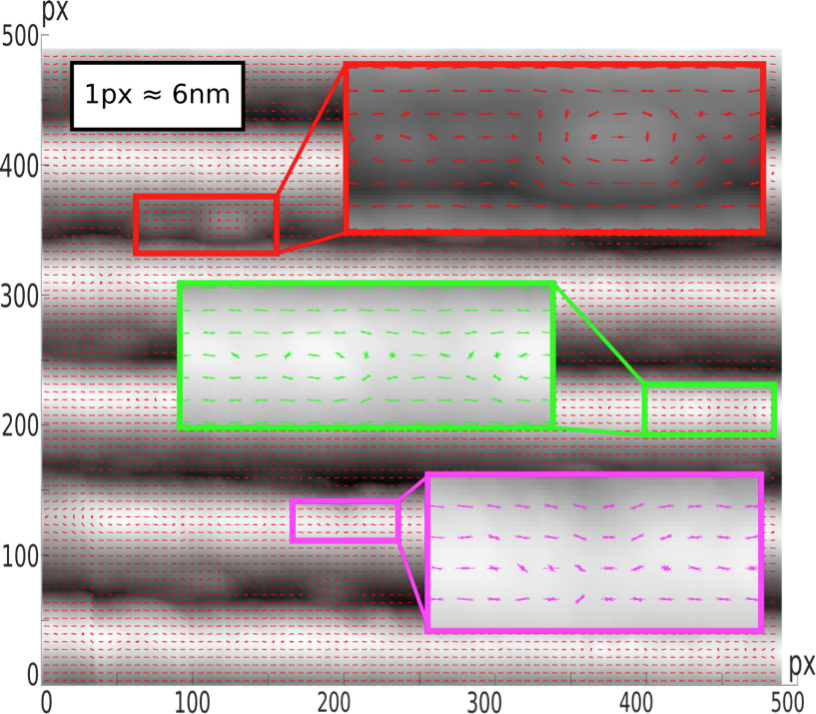
Evaluation of HOG histograms. The example shows a 500 × 500 pixels (about 3 × 3 µm) fragment of an AFM image of a copy of the blazed grating master. Blocks gathering 6 × 6 pixels show the gradients. These are eventually employed to compute the histograms.

Subsequently, the concept of blocks is introduced. Blocks are larger regions of the image containing several cells that allow for the contrast normalization of local histograms. The blocks are used as moving windows that run over the whole image, and include as many histograms as the block contains cells. As the window is sweeping over the image, the same cell contributes to the histogram of different blocks. This procedure acts as an overlapping local contrast that improves the accuracy. HOG is defined as the multicomponent vector that gathers all the histograms obtained from the blocks. An example of block presentation for one of the samples is shown in Figure 7. A 6 × 6 pixels block size has been chosen. A number of lines gathered in each block show the pixel gradients. These are used to calculate the histograms.

The histogram distribution is then assumed to follow a normal Gaussian distribution in angles, centered at 90°. This assumption is obviously not valid for a standard image. In our case, however, the image is a smooth steadily increasing grayscale the gradients of which should point towards the blazing steps; a Gaussian distribution seems appropriate to account for deviations from this preferential direction. The Gaussian FWHM width is employed as a measurement of the gradient dispersion.

## Results and Discussion

**Figure 8:**
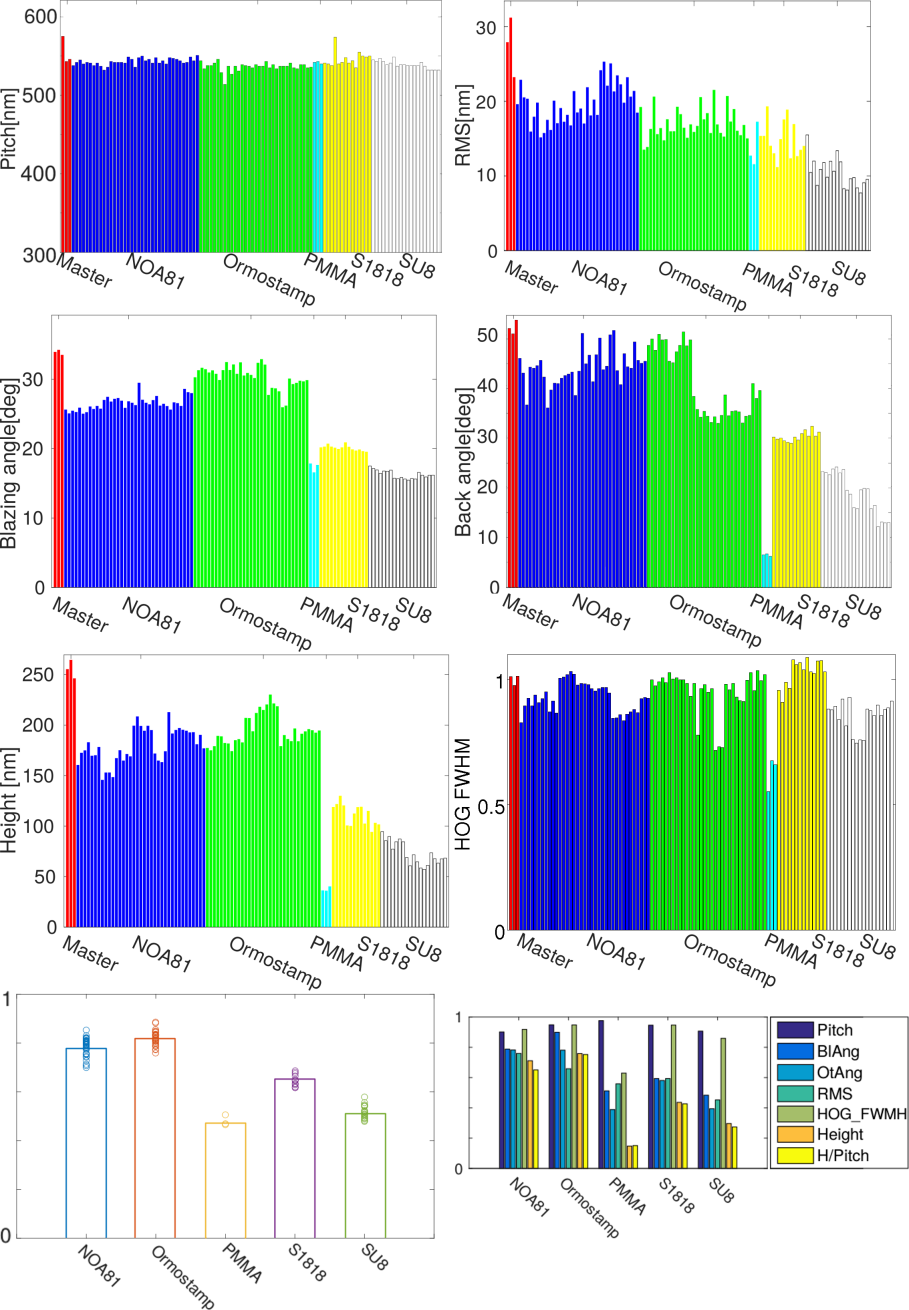
Summary of the results. The sample master and all 103 replica samples are presented. The bottom graphs are averaged figures of merit gathering all parameters (left) and singularizing every parameter (right).

Measurements of the abovementioned parameters in 103 samples are shown in Figure 8. The six upper graphs are histograms of the different parameters; all the samples have been included, along with several measurements of the master at different positions.

The histograms of some parameters are gathered in groups. This is partially due to data processing. Data are obtained from synthetic AFM images that must be properly oriented before averaging. Small angular deviations may lead to biased results. Additionally, the high number of samples precluded applying the same intermediate negative PDMS mold to all the samples: actually, three PDMS molds were used. Moreover, samples were made in batches on different days. Although the experimental procedure was the same, some variables such as room temperature or humidity were not controlled. As a result, some variability between batches was observed, frequently larger than the variability within a single batch. Occasionally, this issue leads to apparently biased measurements, e.g., the back angle of Ormostamp is visibly separated into two families with different average. The result clearly shows that for samples prepared with this manufacturing protocol to be comparable to each other, they must be integrated in the same batch and employ the same negative mold. The height-to-pitch ratio is biased by the same issue. Hence, it has not been included in the results shown in Figure 8.

Despite these difficulties, the results can be employed to explore the limits of the 3D-printed nanoimprint unit, along with the polymers that better suit the unit.

**Pitch** is quite constant and similar in all materials. This is a positive indication about the unit performance and reproducibility. However, it is useless to qualify the different polymers.

Vertical **RMS deviation** apparently shows a high variability, partly due to the large scale of the graph. Actually the deviation of most samples is within 10 nm, which is considered acceptable for the simple custom-made unit tested here. It should be noted that RMS is higher on average for solvent-free materials. This result can be explained assuming that the solvent somewhat flattens the samples diffusing their roughness. The difference is not large, except in a few NOA81 and Ormostamp samples of the same batch, of which the manufacturing protocol was possibly biased.

**Blazing and back angles** clearly show the superior performance of solvent-free materials (NOA81 and Ormostamp) in reproducing the master. Solvents, as commented above, tend to flatten the fresh samples before curing. This has a dramatic effect on the angles. While solvent-free Ormostamp angles are above 90% and NOA81 angles are above 80% of the master angle, materials employing solvents barely reach 50% or less of this angle. This issue is extremely important, since the inability of reproducing these angles can be directly correlated to the lack of nanoimprint reproducibility of any relief features.

A comparable result is obtained for the **height** measurements. Again Ormostamp and NOA81 render a fraction of the master height substantially higher than materials using solvents. The PMMA case is particularly dramatic: the average height is scarcely 15% of the master height.

The FWHM distribution of **HOG gradients** shows some interesting results. The first noteworthy result is the presence of dissimilar groups of values within the same material. We believe that this is a consequence of the extreme sensitivity of HOG to acquisition parameters. HOG is conceived as a descriptor of specific contents in image processing. Data are taken directly from color coordinates or gray levels. Consequently, variations of brightness, contrast or saturation of the image may modify the HOG FWHM distribution making its use as a comparative tool between different materials or even within the same material questionable. Alternative calculation paths are being currently under study. Nevertheless, the issue can be overcome in our case by adjusting manually the image parameters, since our pictures are actually synthetic images rather than photographs. Note that a number of tested samples show HOG values higher than those of the master. This needs not be a sign of better performance; the values are just distribution widths. By now, we will consider that the HOG of a material is better when showing less dispersion and values closer to that of the master. Regarding this benchmark S1818 is the material that performs best. Nevertheless, the number of samples is significantly lower than those of Ormostamp or NOA81; a test with a similar number of samples should be carried out before selecting the best material.

The bottom graphs are figures of merit: an averaged figure of merit including all parameters (bottom left) and a separate set of figures of merit for every parameter (bottom right). As mentioned above, a single figure of merit does not seem the best solution since rather dissimilar parameters are considered. Yet in both cases it is straightforwardly seen that the polymers performing best, according to the tested parameters, are Ormostamp and NOA81. Ormostamp is specially designed for nanoimprint replication; therefore, its superiority can be easily understood. However, the second position of NOA81, only slightly behind Ormostamp, is more surprising. This result can be attributed to the absence of a solvent. Indeed, Ormostamp and NOA81 are the only tested materials that do not require solvent during manufacturing. This subject seems to be crucial for optimum replication, especially regarding parameters in which the vertical dimension is involved, e.g., height and blazing angle.

## Conclusion

A number of parameters have been measured in different materials to ascertain which material performs better in fabrication of nanoimprint replicas. A global figure of merit has been used to establish a tentative ranking. This procedure can be adapted to the requirements of a specific application. This can be done through assigning weights to the different parameters in the figure of merit, or through including other parameters as well as through discarding any unrelated parameter within the application context.

Another important conclusion derived from the results concerns the reliability of the system. The performance of the nanoimprint unit – notwithstanding its fabrication with a 3D printer – can be considered satisfactory, providing the correct material is employed. This opens the opportunity of creating sophisticated nanoimprint elements with good reproducibility using a very simple and inexpensive tool.

## Experimental

A commercial 1800 lines/mm saw-tooth grating (Thorlabs GR13-1850) was chosen as master. Soft NIL was employed for design requirements. Copies were transferred from the original pattern to the target material via a PDMS negative mold. The embossing pressure was regulated through applying vacuum to the system. Details of the nanoimprint unit have been shown elsewhere [7].

The developed fabrication procedure is useful for a relatively small number of samples. Yet parameter dispersion was considered relevant to qualify any material as appropriate; therefore a larger than usual number of samples (more than 100) were prepared. Both, thermally curable and UV-curable materials have been tested: Ormostamp^®^, NOA81, SU8 photoresist, Microposit S1818 photoresist, and poly(methyl methacrylate) (PMMA).

**Ormostamp^®^:** This material is a hybrid polymer provided by Micro Resist Technology. It is specially conceived for nanoimprint replication. Its manipulation is solvent-free and it cures under UV light. The nominal curing wavelength is 355 nm. The material must be deposited on a properly cleaned substrate (glass, Si, SiO_2_). If the resulting thickness is critical, it is advisable to spin-coat the material following the material guidelines [12].

**NOA-81:** It is one of the most popular optical adhesives of the NOA family (Norland). This material is also UV-curable and solvent-free. The nominal curing wavelength is 355 nm. At the first glance, NOA81 is not conceived as a polymer for nanoimprint replication, yet the absence of solvent makes it a good candidate for replicating.

**SU8 2001:** Epoxy-based negative photoresist from MicroChem. It must be spin-coated on a substrate. It employs solvent that must be removed in a prebaking step prior to imprinting. Solvent complicates the manipulation of the material, yet SU8 is attractive because of its ample use for the fabrication of optical microdevices. SU8 is UV-curable and the working wavelength for cross-linking the uncured polymer is 355 nm.

**S1818:** A positive photoresist from Microposit. For this reason, it is not exposed since exposition to UV would ruin the material. Thermal printing NIL is used instead. Solvent has to be used and must be removed prior to imprinting. The curing temperature is 90 °C, the curing time is 5 min on a hotplate before cooling down slowly to room temperature.

**PMMA:** A 5% w/w solution of poly(methyl methacrylate) in chloroform was used. The solution must be spin-coated on a substrate. This material is thermally printed. Once the solution is deposited and the solvent has been removed, the sample is heated up to its glass temperature (105 °C) to obtain a correct imprint.

The pressure applied for the imprinting process was 115 mbar in all cases. The fabrication conditions of the intermediate PDMS mold, including temperature, were kept the same in all the samples.
